# Multi-Omics Analysis of the Effects of *INHA* and *FNDC1* on Clutch Length in Zi Geese

**DOI:** 10.3390/ijms27156798

**Published:** 2026-07-29

**Authors:** Xiuhua Zhao, Shan Yue, Jinyan Sun, Yuanliang Zhang, Fugang Peng, Zhenhua Guo

**Affiliations:** Institute of Animal Husbandry, Heilongjiang Academy of Agricultural Sciences, No. 368 Xuefu Road, Harbin 150086, China

**Keywords:** ceRNA, goose, proteome, molecular docking

## Abstract

Clutch length is a key determinant of egg production in geese and has a direct impact on breeding efficiency. However, the genetic and molecular mechanisms underlying variation in clutch length remain largely unclear in Zi geese. In this study, we integrated whole-genome resequencing with ovarian transcriptomic and proteomic analyses to identify candidate genes and functional single nucleotide polymorphisms (SNPs) associated with clutch length. A total of 200 female Zi geese were monitored throughout a 230-day laying period, from which 20 individuals with the longest clutch length and 20 with the shortest clutch length were selected for multi-omics analyses. Comparative analyses identified 424 differentially expressed genes and 856 differentially expressed proteins between the two groups. Integration of population-differentiated SNPs, genome-wide association study signals, transcriptomic, and proteomic datasets converged on two key candidate genes, *INHA* (inhibin subunit alpha) and *FNDC1* (fibronectin type III domain containing 1). *INHA* expression was negatively associated with clutch length, whereas *FNDC1* expression showed a positive association. Eight missense SNPs were detected across these two genes. Notably, structural modelling and molecular docking analyses demonstrated that the Gly10Cys substitution in INHA markedly increased the binding affinity of INHA homodimerisation (binding energy: −10.4 kcal/mol versus −8.6 kcal/mol for the wild-type protein), promoting the formation of more stable but functionally inactive INHA–INHA homodimers. This finding provides a plausible molecular explanation for the reduced *INHA* mRNA and INHA protein expression observed in geese with longer clutch lengths. These structural analyses suggest that the biological effects of these identified missense SNPs are primarily mediated through protein conformational changes. Collectively, our findings identify *INHA* and *FNDC1* as key regulators of clutch length in Zi geese and reveal a previously unrecognised structural mechanism by which an *INHA* missense variant may influence reproductive performance. These results provide valuable molecular markers and mechanistic insights for the genetic improvement of egg production in geese.

## 1. Introduction

China accounts for approximately 90% of the global production and consumption of domestic geese, and egg-laying performance is one of the key traits determining the economic efficiency of goose production [1]. Compared with chickens, geese exhibit pronounced seasonal reproductive characteristics. Their relatively short laying period and substantial variation in clutch length represent major constraints on the sustainable development of the goose industry [2]. The Zi goose, an elite indigenous breed originating from high-latitude regions, is well known for its excellent egg-laying performance, with an average annual egg production of approximately 90 eggs. However, considerable individual variation in clutch length exists within this breed, and the molecular genetic mechanisms underlying this variation remain largely unexplored [1,3].

Clutch length directly reflects the persistence of egg laying in geese and is an important indicator of laying performance [4]. A longer clutch length indicates more efficient follicular development and a more regular ovulatory rhythm, thereby substantially increasing total egg production [5]. In recent years, with the rapid development of molecular biology technologies, integrative multi-omics analyses have become a powerful approach for elucidating the molecular mechanisms underlying complex reproductive traits in poultry [6,7,8]. The combined application of whole-genome resequencing, transcriptomics, and proteomics enables the systematic identification of trait-associated genetic variants and regulatory pathways across multiple biological levels, including DNA, RNA, and proteins [4,9].

To date, studies investigating the molecular mechanisms underlying egg-laying traits in geese have primarily focused on three aspects: hormonal regulation, ovarian development, and the identification of candidate genes [1,2,3,4]. At the genetic level, genome-wide association studies (GWAS) and selection signature analyses have identified multiple candidate genes associated with egg production and laying period across different goose breeds, including reproduction-related genes (e.g., *AGTR2* and *PCGF6*) and genes involved in retinal photoreception (e.g., *PROS1* and *ZEB2*). These findings suggest that phototransduction-related pathways play important roles in the seasonal regulation of reproduction in geese [1].

The competitive endogenous RNA (ceRNA) regulatory network has emerged as a major focus in studies of reproductive traits in recent years. Circular RNAs (circRNAs) and long non-coding RNAs (lncRNAs) can function as molecular sponges for microRNAs (miRNAs), thereby regulating the expression of their target genes. A previous study investigating ovarian development at different developmental stages in Wanxi White geese constructed a circRNA–miRNA–mRNA regulatory network and identified multiple key ceRNA regulatory axes involved in ovarian development and steroid hormone biosynthesis [10]. However, whether single-nucleotide polymorphisms (SNPs) influence gene expression by altering ceRNA interactions has not yet been systematically investigated in studies of reproductive traits in geese.

In our previous study, analysis of egg-laying records over a 230-day period led to the preliminary identification of 424 differentially expressed mRNAs. Integration with metabolomic data further suggested that clutch length is regulated by a complex polygenic network [3]. Building on these findings, the present study integrated whole-genome resequencing, ovarian transcriptomic, and proteomic data to systematically identify key genes and SNPs associated with clutch length in Zi geese. Furthermore, protein structure modelling, molecular docking, and ceRNA network analyses were performed to characterise the functional effects of the identified SNPs. The objective of this study was to identify the core candidate genes regulating clutch length in Zi geese and to provide a theoretical basis for molecular marker-assisted breeding of high egg-producing goose breeds.

## 2. Results

### 2.1. Comparison of Production Traits

**Figure 1:**
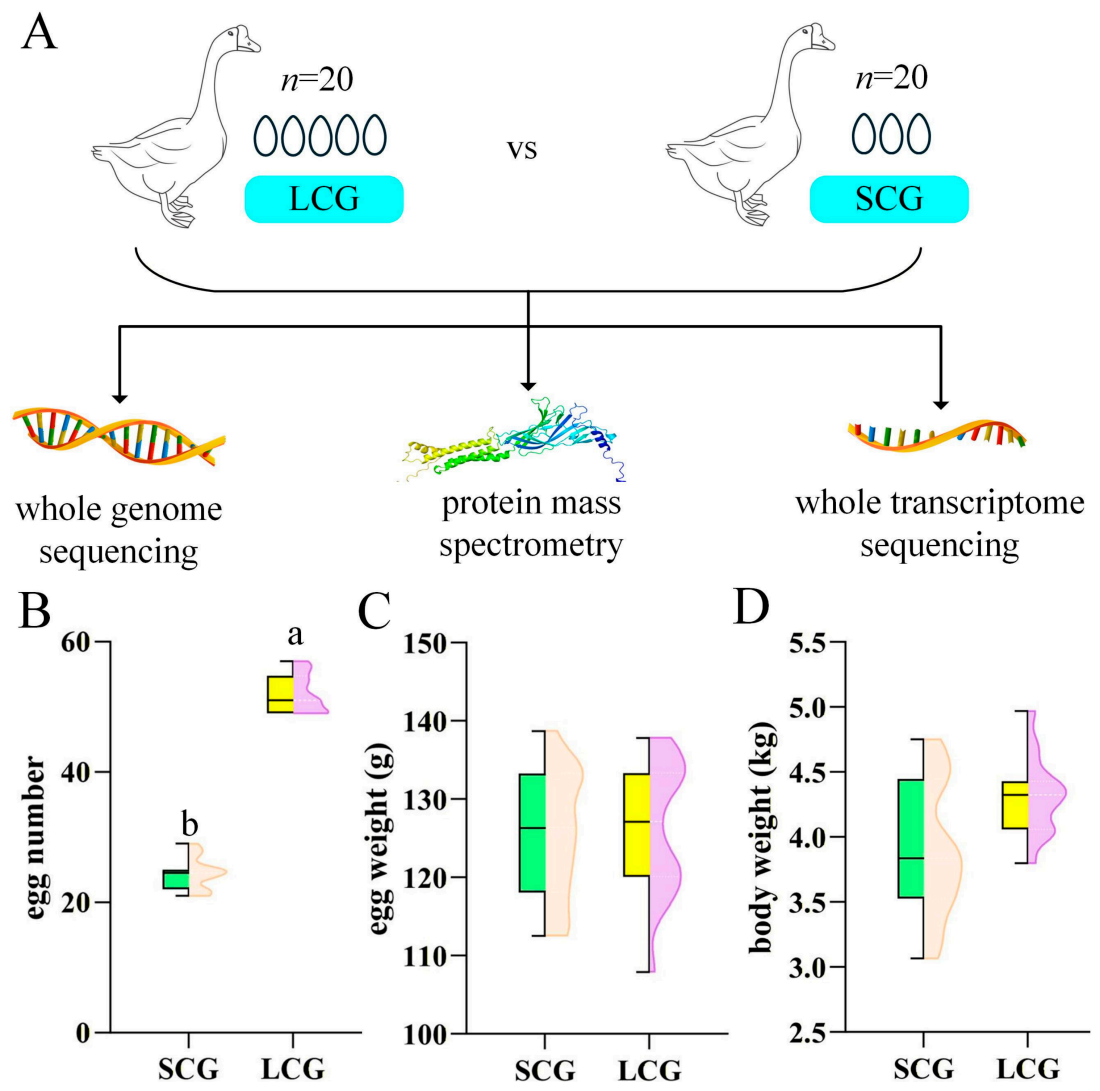
Experimental design and phenotypic comparison between different clutch length groups. (**A**) Experimental design of the present study. (**B**–**D**) Comparison of egg number, egg weight, and body weight between the LCG and SCG (*n* = 40). Different letters indicate significant differences (*p* < 0.05).

As shown in Figure 1B, over the entire 230-day laying period, the long-clutch group (LCG, *n* = 20) exhibited a significantly higher number of eggs (mean = 52 eggs) than the short-clutch group (SCG, *n* = 20, mean = 24 eggs). However, no significant differences were observed in egg weight or body weight between the two groups (Figure 1C,D).

### 2.2. Key SNPs Associated with Different Clutch Lengths

**Figure 2:**
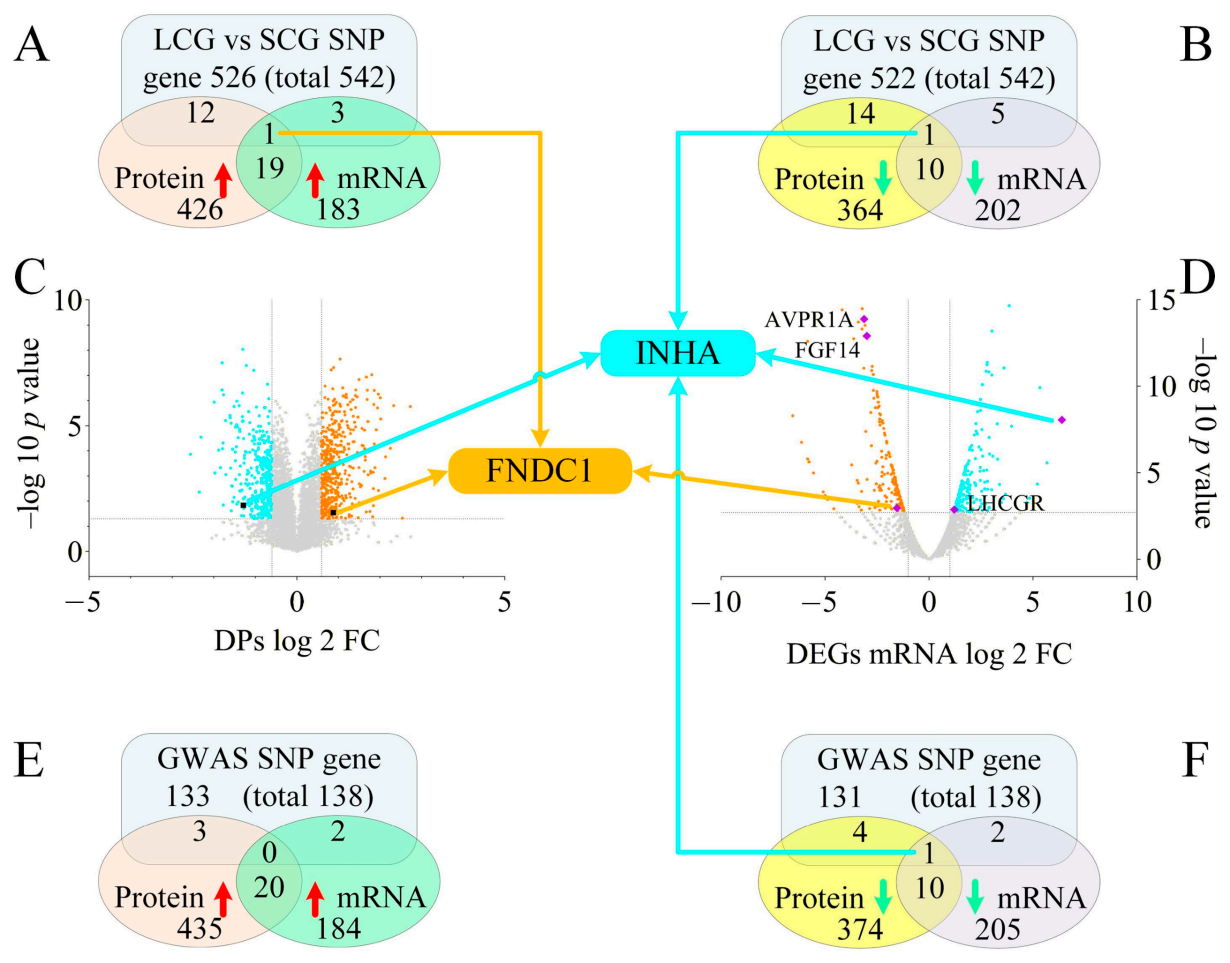
Identification of key SNPs associated with clutch length. (**A**,**B**) Intersection of group-differentiated SNPs with differentially expressed proteins and mRNAs. (**C**,**D**) Volcano plots of differentially abundant proteins and differentially expressed mRNAs. Orange dots indicate upregulated proteins/genes, and blue dots indicate downregulated proteins/genes. (**E**,**F**) Intersection of GWAS-identified SNPs with differentially expressed proteins and mRNAs. Red upward arrows indicate upregulation, and green downward arrows indicate downregulation.

A total of 1360 significant SNPs were identified from the comparison between the LCG and SCG, and these were distributed across 542 genes (Figure 2A,B). Proteomic Principal component analysis (PCA) results (Supplementary Figure S1A) showed clear separation of the LCG and SCG, indicating distinct proteomic profiles. In total, 856 differentially abundant proteins were detected, including 458 upregulated and 398 downregulated proteins (Figure 2C). Additionally, 424 differentially expressed mRNAs were identified, comprising 206 upregulated and 218 downregulated genes (Figure 2D). For the GWAS, 39,827 SNPs were initially identified. Using a significance threshold of *p* < 0.001 (−log10P > 3), 1175 significant SNPs were obtained and annotated to genomic regions including 3′UTRs, 5′UTRs, upstream and downstream regions, and missense variants. These SNPs were distributed across 137 genes (Figure 2E,F). Manhattan and Q–Q plots of the GWAS are shown in Supplementary Figure S1B,C, supporting the reliability of the association results. Integration of differential SNPs with differentially expressed proteins and mRNAs identified *FNDC1* as significantly associated with egg production, with two SNPs located within this gene and showing upregulation in high egg-producing geese. By contrast, *INHA* was identified as a downregulated gene closely associated with egg production and harbouring six SNPs (Table 1).

### 2.3. Pathogenicity Prediction and Higher-Order Structure Modelling of INHA and FNDC1 SNPs

The pathogenicity prediction results for the eight SNPs identified in *INHA* and *FNDC1* are summarised in Table 2. Among these variants, only 61T > C (Trp21Arg) in *INHA* was classified as ‘probably damaging’, whereas the remaining SNPs were predicted to have no significant impact on higher-order protein structure.

**Figure 3:**
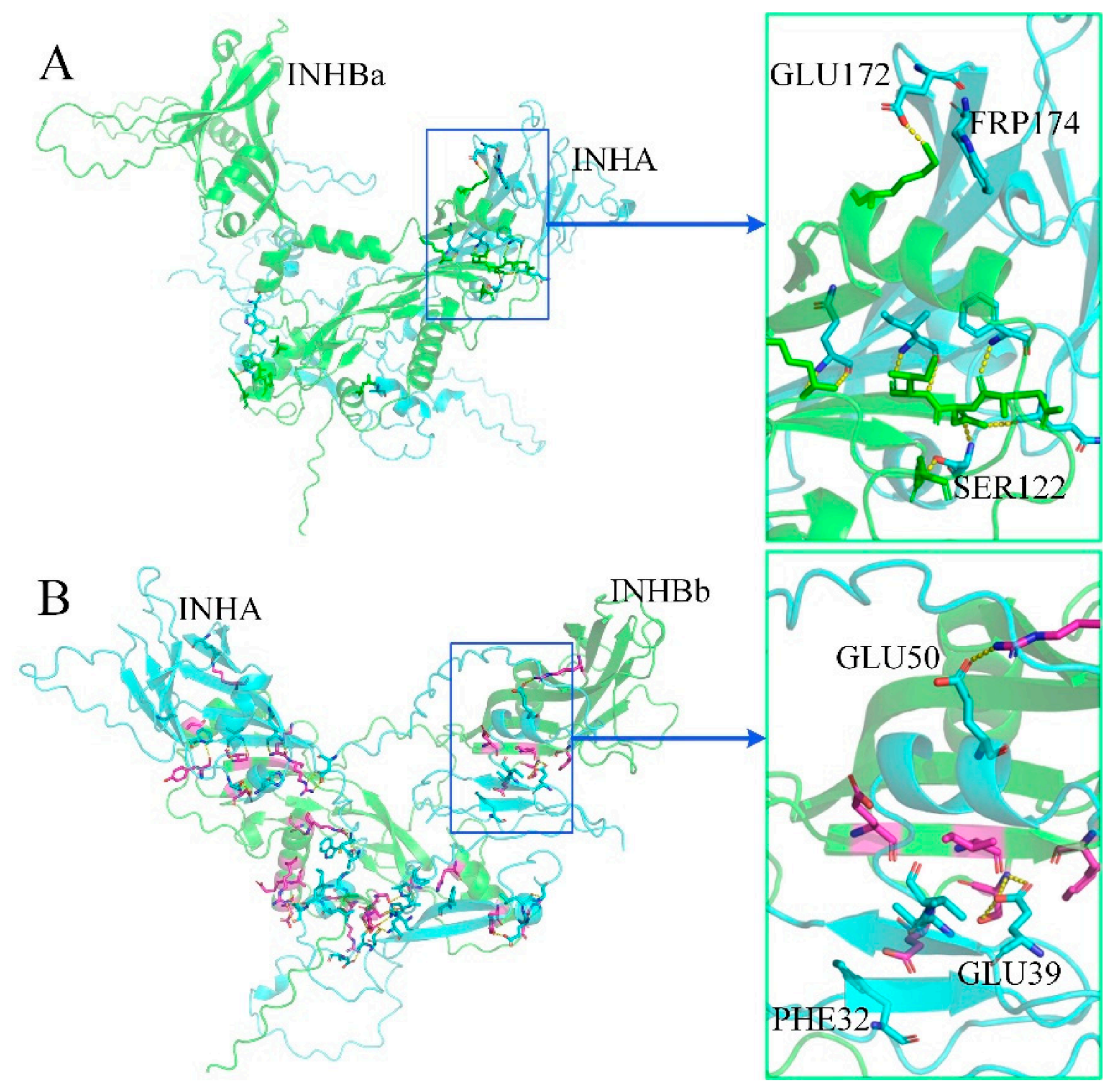
Molecular docking of INHA with INHBa and INHBb. (**A**,**B**) Molecular docking results of INHA with INHBa and INHBb, respectively. INHA is shown as the blue subunit. Residues of INHA within the binding pocket are displayed as blue stick models, indicating interactions at a distance of less than 3 Å. Residues of INHBa and INHBb are shown as green and purple stick models, respectively.

**Figure 4:**
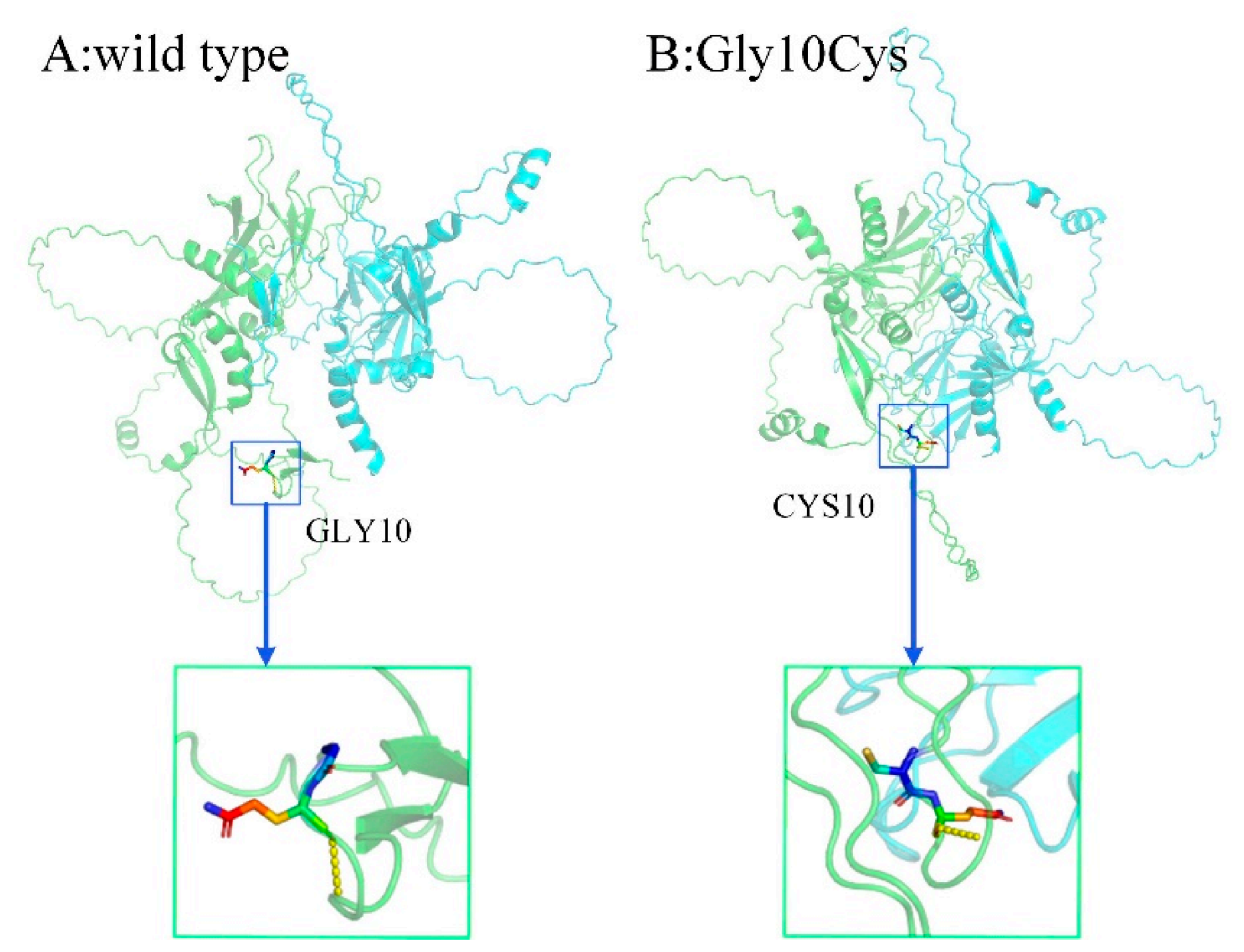
Molecular docking of INHA homodimerization. (**A**,**B**) Molecular docking results of wild-type and Gly10Cys variant INHA homodimerisation. Both the blue and green subunits represent INHA. Residues at position 10 (Gly10 or Cys10) within the binding interface are displayed as stick models, indicating interactions at a distance of less than 3 Å.

Figure 3A,B shows the molecular docking results of INHA with INHBa and INHBb, respectively. No SNP residues were observed within the predicted binding pockets. Table 3 summarises the binding energy changes associated with the different SNP-induced amino acid substitutions. Overall, no substantial alterations in docking affinity were observed for INHA interactions with INHBa or INHBb. By contrast, analysis of INHA homodimerisation revealed that the Gly10Cys variant exhibited a higher binding affinity (−10.4 kcal/mol) than the wild-type protein (−8.6 kcal/mol), corresponding to an approximately 20% increase in binding strength. Structural visualisation (Figure 4A,B) further demonstrated that residue Cys10 is located at a key interface region of the dimer, at a distance of less than 3 Å from the interaction interface.

### 2.4. INHA- and FNDC1-Related ceRNA Interactions

**Figure 5:**
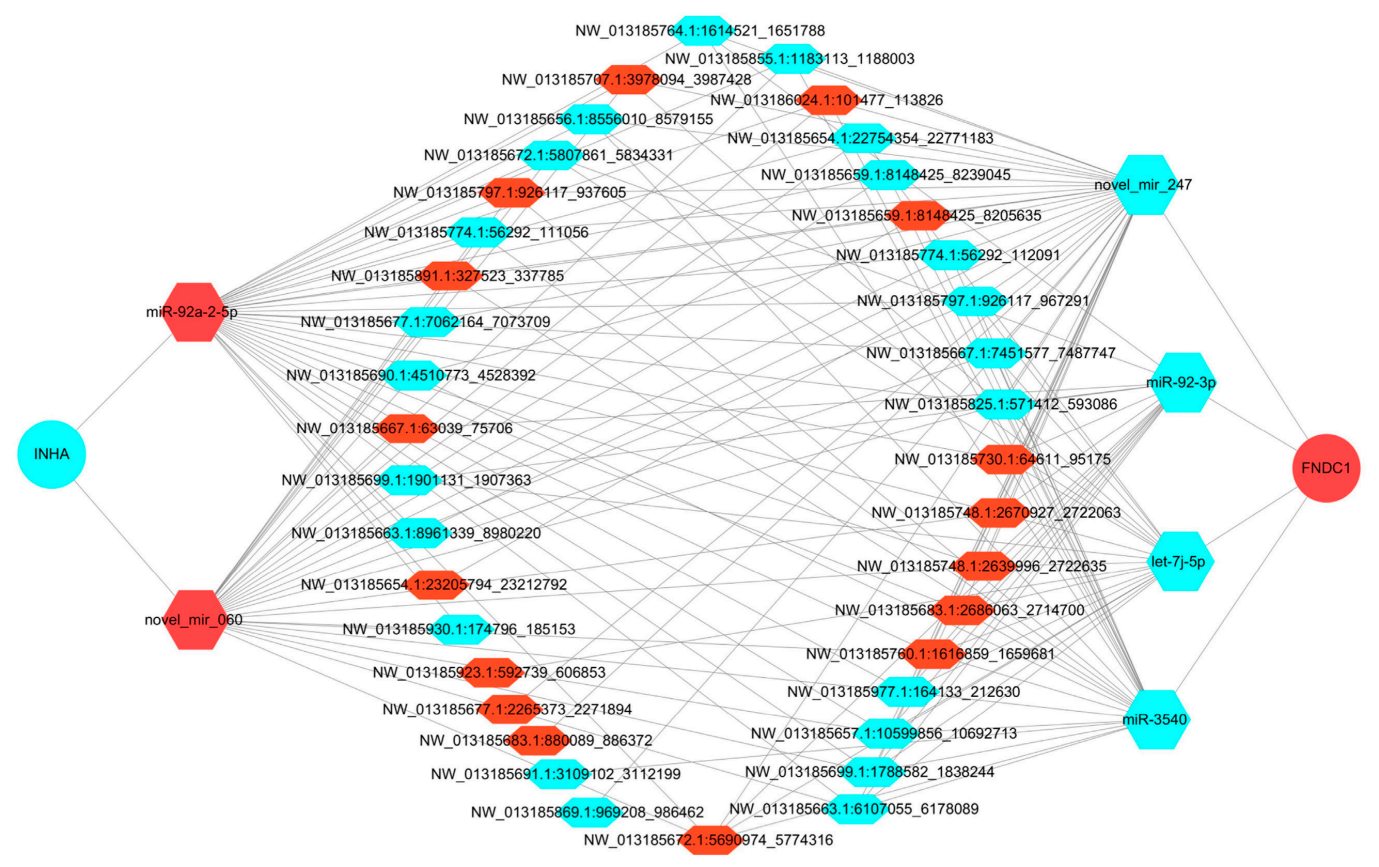
ceRNA regulatory network of *INHA* and *FNDC1*. The network illustrates miRNA–mRNA and circRNA–miRNA regulatory relationships, in which miRNAs suppress mRNA expression and circRNAs act as molecular sponges for miRNAs. Red nodes indicate upregulated expression, whereas blue nodes indicate downregulated expression.

A ceRNA regulatory network involving *INHA* and *FNDC1* was constructed (Figure 5). In silico analysis further predicted an interaction between *INHA* mRNA and the differentially expressed miRNA miR-92a-2-5p (Supplementary Figure S3). However, none of the eight SNPs identified in *INHA* and *FNDC1* were predicted to affect the binding between the mRNAs and the differentially expressed miRNA.

## 3. Discussion

In this study, two genes (*INHA* and *FNDC1*) exhibited consistent and significant changes at both the mRNA and protein levels (Figure 2), suggesting that they may serve as candidate genes for the genetic improvement of egg production in geese.

### 3.1. Role of INHA in Reproductive Regulation

In the present study, both *INHA* mRNA and protein levels were significantly decreased in the LCG compared with the SCG. *INHA* is a key gene involved in the regulation of reproductive function and encodes the α-subunit of inhibin, which forms a functional inhibin complex through dimerisation with INHBa or INHBb [11,12]. In female geese, inhibin is primarily secreted by ovarian granulosa cells and regulates reproductive function through endocrine, paracrine, and autocrine mechanisms, particularly via its action on the pituitary gland. It suppresses follicle-stimulating hormone (FSH) secretion, thereby inhibiting follicular development and ovulation [13]. Previous studies have also reported that SNPs in *INHA* are significantly associated with litter size in sheep and goats [13,14,15,16] and that *INHA* is involved in the regulation of ovulation in mice, hens, and cattle [12,17,18,19]. In addition, inhibin A has been reported to participate in feedback regulation by suppressing *INHA* mRNA expression [20]. Therefore, in the present study, we initially attempted to determine whether SNPs might affect the higher-order structural properties of the protein, thereby influencing pituitary–ovarian feedback regulation. However, no SNPs were found to be directly involved in the protein–protein interactions of INHA (Figure 3).

### 3.2. FNDC1-Mediated Regulation of Sex Hormones

In a previous study on skeletal muscle regeneration in mice, FNDC1 was identified as binding to integrin β1 [21]. In the present study, we also performed molecular docking simulations to investigate this interaction (Supplementary Figure S2). *FNDC1* is generally considered a biomarker in cancer research [22,23]. In a study of prostate cancer, *FNDC1* was shown to regulate androgen receptor expression [24]. Using supervised machine learning and gene network modelling approaches, *FNDC1* was also identified as a candidate biomarker for pregnancy status in dairy cattle [25]. Moreover, *FNDC1* has been reported to participate in the regulation of reproduction in Small Tail Han sheep by modulating the pituitary–ovarian axis and influencing litter size [26]. Although there are no clear reports in goose ovaries, based on our multi-omics data showing its positive association with clutch length, we speculate *FNDC1* might play a beneficial role in ovarian follicular development, which warrants further investigation (Figure 2).

### 3.3. SNP-Induced Alterations in the Higher-Order Structures of INHA Proteins

Given the large number of SNPs that may lead to missense mutations, computational simulation approaches provide an efficient strategy for rapidly prioritising candidate SNPs prior to experimental validation [27,28]. In the present study, we similarly employed in silico approaches to evaluate the effects of SNPs on higher-order protein structure and molecular docking. The results suggested that several SNPs may alter protein conformation and potentially exert deleterious effects (Table 2 and Table 3). Notably, an unexpected finding was that the Gly10Cys variant in INHA enhanced homodimerisation affinity, leading to the formation of a more stable INHA–INHA homodimer (Figure 4). This may result in a substantial proportion of INHA protein existing in a homodimeric form that lacks biological activity. Under physiological conditions, INHA forms heterodimers with INHBa or INHBb to generate functional inhibin, which suppresses FSH secretion. By contrast, the Gly10Cys substitution may promote the formation of non-functional INHA homodimers, thereby reducing the availability of functional heterodimers and weakening FSH inhibition. This could accelerate follicular development and consequently extend clutch length. Furthermore, we propose a hypothetical regulatory model in which a threshold level of functional wild-type INHA protein is required to activate pituitary–ovarian feedback regulation. In individuals carrying the Gly10Cys variant, although overall *INHA* mRNA and protein levels are reduced, a substantial proportion of the translated protein may be sequestered into non-functional homodimers. As a result, despite reduced functional protein output, the feedback regulation axis may not be properly activated. This may contribute to the observed simultaneous downregulation of both *INHA* mRNA and protein in Gly10Cys carriers.

### 3.4. SNP-Mediated Regulation of ceRNA Networks

Another proposed mechanism suggests that SNPs may regulate mRNA translation into protein through ceRNA networks [29,30]. In a study involving 1140 Liaoning cashmere goats, a ceRNA regulatory network constructed after SNP screening indicated that miR-93 may negatively regulate cashmere fineness in individuals carrying the G1355A variant [31]. In addition, by retrieving SNP and differentially expressed gene data from human inflammatory bowel disease databases, a bioinformatics-based study indirectly suggested that SNPs in *PTPN2* may participate in ceRNA-mediated regulation [32]. Using a similar strategy, another study reported that the expression of *SURF4* is influenced by the rs550057 SNP and may be involved in regulating lung cancer progression [33]. In the present study, we also investigated the relationship between SNPs and the ceRNA regulatory network (Supplementary Figure S3). Given that all eight identified SNPs are missense variants located within the coding regions, it is biologically expected that they do not overlap with specific miRNA binding sites, which are predominantly located in untranslated regions (UTRs) or intronic sequences. Consequently, our analysis showed no evidence that these specific coding-region SNPs affect miRNA–mRNA interactions. However, this expected outcome does not exclude the involvement of ceRNA regulation in clutch length variation. Regulatory effects are highly likely to occur through other unanalyzed SNPs located in UTRs or intronic regions. Moreover, ceRNA regulation is thought to be mediated predominantly by lncRNAs and circRNAs rather than SNPs within mRNA coding regions. Therefore, future studies should expand the scope of analysis to include a broader range of genomic variants and ceRNA axes associated with clutch length regulation, rather than being limited to *INHA*- and *FNDC1*-centred networks.

### 3.5. Potential Effects of Synonymous SNPs

In humans, synonymous SNPs have been reported to contribute to disease susceptibility, and several databases have been established to curate potentially deleterious synonymous variants, including HGMD (http://www.hgmd.org, 17 May 2025) [34], ClinVar (https://www.ncbi.nlm.nih.gov/clinvar/, 17 May 2025) [35], and dbDSM (https://bioinfo.ahu.edu.cn:8080/dbDSM/index.jsp, 17 May 2025) [36]. Accumulating evidence suggests that synonymous mutations are not necessarily neutral because they may involve codon substitution bias, and the presence of rare codons can disrupt translational efficiency and accuracy. Therefore, synonymous variants may exert functional effects by altering codon usage bias [37,38]. Given the limited availability of annotated genomic resources in geese, synonymous SNPs were not investigated further in the present study. In addition, variants located in intronic regions, particularly promoter-associated SNPs, were not systematically analysed. Future studies will address these limitations by performing functional validation of intronic SNPs using luciferase reporter assays based on primary ovarian granulosa cell cultures. Moreover, larger population cohorts will be included in future studies to comprehensively identify SNPs associated with egg-laying traits.

### 3.6. Limitation

In Figure 2, it can be seen that there are fewer than 20 genes that conform to mRNA and protein co-expression, and the screening of SNPs is only to narrow down the scope of research objectives. When processing SNP data in this study, there was a lack of consideration for the risk of false positives that may arise from not performing multiple test correction (FDR). Therefore, the summarized differential SNPs may have false positives and certain limitations. No missense mutations were found to affect ceRNA regulation because regulatory SNPs that affect binding are more likely to exist in the UTR or intron regions. However, such results were not validated in vitro/in vivo experiments in this study (e.g., co immunolocalization of mutant and wild type proteins, assessment of FSH secretion in primary cell cultures with the addition of mutant inhibition), which is a limitation that this study did not cover.

### 3.7. Conclusions

This study successfully integrated multiple omics data and identified *INHA* and *FNDC1* as key candidate genes regulating the clutch length of egg production in geese, making a substantial contribution to understanding and improving the molecular genetic basis of goose reproductive traits. The core innovation of this study lies in proposing a biologically plausible mechanism hypothesis, which provides a novel protein structural explanation for the downregulation of *INHA* expression levels in high-yielding geese. However, due to the fact that the structural model and molecular mechanism findings in this study are mainly based on computer simulations (in silico), caution must be exercised in interpreting these predictive results. If the genetic markers and molecular mechanisms proposed in this article are to be truly applied to precision breeding of high-yield poultry, they must first be confirmed through rigorous in vivo and in vitro functional experiments.

## 4. Methods

### 4.1. Ethics of Experimental Animals

This research was approved by the Committee for Animal Welfare at the Institute of Animal Husbandry, Heilongjiang Academy of Agricultural Sciences (HAAS) (No. NKYXMS-20240117, 17 January 2024), in accordance with the Laboratory Animal Guidelines for Ethical Review of Animal Welfare (GB/T 35892-2018).

### 4.2. Determination of Egg-Laying Traits in Geese

A total of 200 healthy female geese of similar age, maintained under identical feeding and management conditions, were housed individually in cages. Egg-laying performance was monitored throughout the entire laying period (230 days, from the onset to the end of egg laying). The onset and cessation of egg laying were recorded for each goose [3]. Based on clutch length, the geese were classified into a long-clutch group (LCG) and a short-clutch group (SCG). Among the 25 geese with the longest clutch lengths and the 25 with the shortest clutch lengths, 20 individuals were randomly selected from each group for subsequent analyses (Figure 1A). Egg number, egg weight, and body weight were compared between the two groups as a quantitative trait. Statistical differences in these phenotypic traits were evaluated using one-way analysis of variance (*p* < 0.05).

### 4.3. Whole-Genome Resequencing and Identification of Candidate Genes

After completion of the egg-laying period, laying performance traits were determined, and 20 high-producing geese in the LCG and 20 low-producing geese in the SCG were identified. Blood samples were collected from all individuals for genomic DNA extraction, and whole-genome sequencing was performed at an average depth of approximately 10 Gb per sample. After obtaining clean reads, the sequences were aligned to the reference genome, followed by population genetic analyses and genome-wide selection signature analyses to identify candidate selective regions associated with egg production and to annotate genes potentially related to laying performance.

Specifically, SNPs were identified using two complementary approaches. In the first approach, the LCG and SCG were treated as two separate groups, and Fisher’s exact test was applied to detect group-differentiated SNPs (*n* = 40). SNPs with *p* < 0.05 were considered significantly different between groups. In the second approach, SNP datasets from all 40 individuals were combined, and a GWAS was performed using clutch length as the phenotype (*n* = 40). SNPs with *p* < 0.001 (−log10P > 3) were considered significant. Significant SNPs were annotated to genomic regions, including 3′UTRs, 5′UTRs, upstream and downstream regions, and missense variants. Genes harbouring these variants were intersected with differentially expressed mRNAs and differentially abundant proteins to identify overlapping candidate genes. SNPs located within these integrated candidate genes were ultimately defined as putative functional SNPs associated with egg-laying performance.

### 4.4. Ovarian Transcriptome Sequencing and Differential Gene Expression Analysis

After completion of the egg-laying period, reproductive phenotypes were determined, and ovarian tissues were collected from three high egg-producing geese and three low egg-producing geese for whole-transcriptome sequencing [3]. mRNA, lncRNA, circRNA, and miRNA sequencing datasets were obtained. For mRNA differential expression analysis, genes with an absolute fold change (|FC|) of >2 (i.e., upregulated >2-fold or downregulated <0.5-fold) and *p* < 0.002 (Student’s *t*-test) were considered significantly differentially expressed. This threshold corresponds to an absolute log2 FC (|log2FC|) of ≥1. The same criteria were applied to the identification of differentially expressed circRNAs. For miRNA analysis, differentially expressed miRNAs were defined as those with *p* < 0.05 and mean read counts of ≥10.

### 4.5. Quantitative Proteomic Analysis of Ovarian Tissues

In addition, ovarian tissues from three high egg-producing geese and three low egg-producing geese (as described above) were subjected to quantitative proteomic analysis. Principal component analysis (PCA) was first performed to assess global differences between the two groups, which indicated clear separation of the high- and low-production groups. Differentially abundant proteins were then identified using a FC threshold of >1.5 (i.e., upregulated >1.5-fold or downregulated <0.67-fold) and *p* < 0.05 (Student’s *t*-test). This corresponds to |log2FC| ≥ 0.587. Based on these criteria, the numbers of upregulated and downregulated proteins in the two groups were determined.

### 4.6. Identification of Key SNPs Associated with Clutch Length

SNPs identified from the comparison between the high egg-producing group (LCG; *n* = 20) and the low egg-producing group (SCG; *n* = 20) were intersected with differentially expressed genes showing consistent upregulation or downregulation, as well as differentially abundant proteins, to obtain a set of candidate SNPs. Similarly, SNPs identified through GWAS were subjected to the same integrative intersection strategy to further refine the list of candidate SNPs.

### 4.7. Pathogenicity Prediction and Higher-Order Structural Modelling of Variant Proteins

After validation of candidate SNPs, the sequences of *INHA* (XM_013171136.1) and *FNDC1* (XM_013186804.1) were retrieved from the National Center for Biotechnology Information (Bethesda, MD, USA). Amino acid substitutions corresponding to the identified mutation sites were introduced, and the potential functional impacts of the variants were evaluated using PROVEAN (http://provean.jcvi.org, 27 May 2025) and PolyPhen-2 (https://genetics.bwh.harvard.edu/pph2/, 27 May 2025).

For *INHA*, six SNP-induced amino acid substitutions were analysed. Protein structures were predicted using AlphaFold 3 (https://alphafoldserver.com, 27 May 2025), followed by molecular docking with INHBa (XP_066848292.1). The resulting complexes were used to calculate binding energies using PRODIGY (https://wenmr.science.uu.nl/prodigy, 27 May 2025). Structural visualisation of protein–protein docking results was performed using PyMOL software v3.1 (www.pymol.org, 27 May 2025). The same procedure was applied to INHA variants docked with INHBb (XP_047928155.1). For *FNDC1*, two SNP-induced amino acid substitutions were analysed and docked with integrin beta-1 (XP_047932552.1) using the same workflow. In addition, INHA variants carrying the six SNP-induced amino acid substitutions were further subjected to homodimerisation docking analysis of INHA–INHA interactions using the same computational pipeline.

### 4.8. ceRNA Regulatory Network of Candidate SNPs

For newly identified circRNAs, miRNA target prediction was performed using both miRanda (v3.3a) and TargetScan (http://www.targetscan.org/, 9 May 2025). The binding interactions between circRNAs and miRNAs were predicted using a miRanda free energy threshold of ≤−20 and the default parameters in TargetScan. Only interactions supported by both tools (i.e., the intersection of the two prediction results) were retained. For lncRNA target gene prediction, targets were classified into cis- and trans-regulatory modes. In the cis analysis, protein-coding genes located within 20 kb upstream or downstream of each lncRNA were considered potential targets. For genes beyond this range, trans-regulatory relationships were inferred based on expression correlation analysis between lncRNAs and mRNAs. A Pearson correlation coefficient of |r| ≥ 0.9 was used as the cut-off for identifying significant lncRNA–mRNA associations. miRNA target genes of mRNAs were predicted using the Weishengxin platform (http://www.bioinformatics.com.cn, 5 May 2025). In addition, SNP-containing sequences in *INHA* and *FNDC1* were incorporated to assess their potential effects on binding to differentially expressed miRNAs, based on miRNA–mRNA interaction predictions. The ceRNA network was visualized using Cytoscape software (version 3.10.2, https://cytoscape.org/, 5 May 2025).

## Figures and Tables

**Table 1:** Key missense SNPs associated with clutch length.

	CHROM	Gene ID	Transcript ID	Codon Mutation	Amino Acid Substitution
**1**	NW_013185660.1	*INHA*	XM_013171136.1	c.28G > T	p.Gly10Cys
**2**				c.61T > C	p.Trp21Arg
**3**				c.506G > A	p.Arg169Gln
**4**				c.664A > G	p.Ser222Gly
**5**				c.739C > G	p.Leu247Val
**6**				c.1031T > C	p.Leu344Pro
**7**	NW_013185733.1	*FNDC1*	XM_013186804.1	c.71C > T	p.Ser24Leu
**8**				c.38C > T	p.Thr13Met

**Table 2:** Pathogenicity prediction of missense SNPs.

	Gene ID	Amino Acid Substitution	PROVEAN Prediction	PROVEAN Score	PolyPhen-2 Results	PolyPhen-2 Score
**1**	*INHA*	p.Gly10Cys	Neutral	−0.152	NA *	NA
**2**		p.Trp21Arg	Neutral	0.425	Probably damaging	0.974
**3**		p.Arg169Gln	Neutral	1.118	Benign	0.009
**4**		p.Ser222Gly	Neutral	−0.128	Benign	0.001
**5**		p.Leu247Val	Neutral	0.158	Benign	0.027
**6**		p.Leu344Pro	Neutral	−0.033	NA	NA
**7**	*FNDC1*	p.Ser24Leu	Neutral	−0.152	NA	NA
**8**		p.Thr13Met	Neutral	−0.039	NA	NA

* PolyPhen-2 score not available.

**Table 3:** Binding energy evaluation of SNP-containing protein complexes.

	Protein Complex	Amino Acid Substitution	ΔG (kcal/mol)	Charged–Charged	Charged–Polar	Polar–Polar
**1**	INHA–INHBa	Wild type	−19.1	23	17	7
**2**		Gly10Cys	−21.1	22	24	10
**3**		Trp21Arg	−23.5	24	22	5
**4**		Arg169Gln	−24.5	26	26	8
**5**		Ser222Gly	−21.3	20	33	6
**6**		Leu247Val	−23.1	23	17	9
**7**	INHA–INHBb	Wild type	−28.5	18	34	5
**8**		Gly10Cys	−25.2	21	36	6
**9**		Trp21Arg	−26	15	28	5
**10**		Arg169Gln	−27.2	16	28	4
**11**		Ser222Gly	−22.9	15	27	9
**12**		Leu247Val	−28.2	27	35	5
**13**	INHA–INHA	Wild type	−8.6	5	18	7
**14**		Gly10Cys	−10.4	18	10	12

## Data Availability

The original contributions presented in this study are included in the article/Supplementary Materials. Further inquiries can be directed to the corresponding authors.

## Supplementary Materials

The following supporting information can be downloaded at: https://www.mdpi.com/article/10.3390/ijms27156798/s1.
